# In Vitro Studies concerning Selected Properties of a Composite Material Blended with Nanofluoroapatite Crystals

**DOI:** 10.3390/ma14237295

**Published:** 2021-11-29

**Authors:** Marta Zietek, Maciej Dobrzynski, Katarzyna Fita, Dorota Diakowska, Adam Watras, Rafal Jakub Wiglusz

**Affiliations:** 1 Department of Pediatric Dentistry and Preclinical Dentistry, Wroclaw Medical University, Krakowska 26, 50-425 Wroclaw, Poland; marta.zietek@umed.wroc.pl (M.Z.); katarzyna.fita@umed.wroc.pl (K.F.); 2 Department of Basic Sciences, Wroclaw Medical University, Bartla 5, 51-618 Wroclaw, Poland; dorota.diakowska@umed.wroc.pl; 3 Institute of Low Temperature and Structure Research, Polish Academy of Sciences, Okolna 2, 50-422 Wroclaw, Poland

**Keywords:** nanomaterials, nanofluoroapatite, caries prevention

## Abstract

The aim of the paper was to determine the potential for fluorine release from an original composite material blended with nanofluoroapatite (FAp). The level of fluoride ion emission into deionized water and saline was studied over a period of 12 weeks. Values were recorded after 1, 3, 24, 48, 72, and 96 h and then weekly for a period of 12 weeks. There were statistically significant differences in the periods of fluoride ion release from 5%FAp and 2%FAp materials into saline solution as well as into deionized water. The highest fluorine release from 5%FAp + polymer was observed in the 10th and 11th week of incubation (for saline solution) and in the 9th, 10th, and 11th week (for deionized water). The highest fluorine release from 2%FAp + polymer was observed in the 9th, 11th, and 12th week of incubation for both environments. Total fluoride ion release from 5%FAp + polymer and mean fluoride release levels were similar in 5%FAp and 2%FAp in both environments. Both tested materials (5%FAp and 2%FAp) show the ability to release fluoride ions over a long time in the experimental environment.

## 1. Introduction

The main objective of dentistry is caring for the health of the oral tissue. Caries is the most common chronic disease of the oral cavity. It is considered a civilization disease. According to the definition of the World Health Organization (WHO), it is a local pathological process that leads to decalcification of the enamel and dentin, decomposition of hard dental tissue, and, consequently, the formation of a dental cavity. When untreated, caries can affect chewing, speech, and the quality of the patient’s life [1,2].

Prevention and early detection of caries as well as the treatment of carious lesions are constant challenges. While treatment of caries and its consequences is often complicated and expensive, its prevention is simple, less expensive, and effective [2].

Fluorides, which reduce acid production from plaque bacteria by building into the apatite of the enamel, play a key role in the prevention of caries. Since constant, long-term supply of low concentrations of fluorine to the oral environment is most beneficial from the perspective of caries prevention, the use of materials releasing fluoride ions has become a common practice in dentistry. On the one hand, these materials release fluoride—during the hardening reaction as well as for several years after the placement of the filling—and on the other, they absorb fluoride from its exogenous supply (e.g., toothpastes, fluorine rinses, and food), thus becoming a specific fluorine reservoir for the oral environment [3,4,5,6,7]. Studies show that the release of fluoride ions from glass ionomer cements lasts for 2.7–8 years, and from composite materials up to even 5 years [8,9].

In restorative and regenerative dentistry involving the hard tissues of the tooth and the alveolar bone, great importance is attached to antibacterial and osteoconductive properties of biomaterials [10,11]. Antibacterial properties are related to the doping of biomaterials with different compounds such as fluoride, silver, or copper nanoparticles [11,12,13]. The antibacterial effect of fluoride is caused by the direct inhibition of cellular enzymes (in combination with metals or directly). Moreover, fluoride enhances the proton permeability of cell membranes as hydrogen fluoride (HF) [14].

“Passive” dental materials have been replaced by “smart materials” that can respond to an external stimulus, such as pH, temperature, moisture, light, chemicals, an electric field, or a magnetic field, by changing their shape, color, or size or releasing ions of the desired elements [15].

Over the years, scientists have proposed many formulations, varying in terms of their ingredients, structural properties, bioactive properties, or manufacturing technology; however, biocomposites controlled at the atomic or molecular level appear to be the most promising ones.

Nanotechnology, considered to be the result of the rationalization of science and technology, has become one of the most preferred technologies in medical applications, including dentistry. An important contribution of dental nanomaterials is the identification of oral health problems through improved diagnostic evaluation and treatment of dental diseases with the use of bionanomaterials [16,17]. The first definition of “nanotechnology” was formulated in 1974, in a paper by Norio Taniguchi (Tokyo Science University), according to which “nanotechnology” consisted of the processing, separation, consolidation, and deformation of materials by a single atom or molecule.

The term “nanomedicine,” on the other hand, was first used by Freitas in the year 1993 and was defined as observing, controlling, and treating the biological systems of the human body at the molecular level with the use of nanostructures and nanodevices.

The definition of nanotechnology, however, does not constitute its beginning. The concept of nanotechnology was already formulated in 1959 by physicist Richard Feynman. He presented it at the conference of the American Physical Society at the California Institute of Technology, which was also held in 1959. While Feynman did not use the term “nanotechnology” or “nanoscience”, he described a process in which a scientist could manipulate materials at the atomic or molecular level.

In the search for the ideal material, we increasingly look for materials that restore the lost tissue, are biocompatible and durable, and at the same time have a preventive effect on the hard tissues of the tooth. In recent years, dental materials science has also incorporated the technology of nanomaterial production; one could say that it has actually been revolutionized by it, so much so that nanotechnology itself has become the most popular field of research, covering a wide range of applications in dentistry [18].

Thus, nanomaterials are used today in the treatment of not only caries, but also dentine hypersensitivity and remineralization of dental hard tissues, as well as the prevention of biofilm formation and the prevention of dental caries progression [16,19].

The aim of the study was to determine the fluorine release capacity of an original composite material blended with a different content of nanofluoroapatite (FAp, 2 or 5%) over a period of 12 weeks in two types of solutions—saline and deionized water.

## 2. Materials and Methods

Materials used in the research:

- i-FLOW (i-dental, Siauliai, Lithuania): a light-curing, nano, flowable, radiopaque composite, under the Vita shades, based on Barium glass filler and EOBPADMA, UDMA, Bis-GMA, TEGDMA resins.
- Nanofluoroapatite: The nanocrystalline powder of fluorapatite was prepared by a co-precipitation method. Analytical-grade Ca(NO_3_)_2_·4H_2_O (99+%, Acros Organics, Geel, Belgium), NH_4_H_2_PO_4_ (99.995%, Alfa Aesar, Haverhill, MA, USA), NH_4_F (98%, Alfa Aesar, Haverhill, MA, USA), and NH_3_ ·H_2_O (99%, Avantor, Gliwice, Poland) were used as starting substrates and for pH adjustment. Subsequently, calcium nitrate was dissolved in deionized water, and then suitable amounts of ammonium phosphate dibasic and then ammonium fluoride were added to the mixture leading to fast precipitation of the by-product. The pH of the dispersion was modulated to 8–9 by adding ammonia, then the suspension was heated and stirred on a stirring plate for 3 h. Finally, the obtained product was centrifuged, rinsed with de-ionized water several times, dried at 70 °C for 24 h, and heat-treated at 450 °C for 6 h to obtain nanofluoroapatites.

Samples of the materials were prepared in the form of a pellet, 5 mm in diameter and 2 mm thick, and were made in the shape of cylinders using a polyethylene matrix. The mass of samples was about 55 mg. For the 2%FAp sample, 1.1 mg of Fap was used, while for the 5%Fap sample, 2.75 mg of FAp was used. The samples were fabricated with the use of the LED Elipar II lamp (3M ESPE, St. Paul, MN, USA) emitting light in the wavelength range of 400–515 nm with a maximum intensity of 800 mW cm^−2^. After curing, the samples were polished and conditioned, corresponding to the regular protocol in a clinical setting. Their contact area was calculated. Subsequently, they were immersed in the studied solutions and left without stirring in closed containers at 37 °C for a suitable period to determine the fluoride release from the materials. Five samples of each material were prepared for each environment (total n = 20). Each sample was examined three times, and an average value was calculated based on the three results. The release of fluoride ions from these materials into a saline solution (0.9% NaCl) and deionized water was tested for 12 weeks. The ion-selective ORION electrode model 9609 (Thermo Fisher Scientific Co., Waltham, MA, USA) and a microcomputer pH/ionometer CPI-551 Elmetron were used for measurements. The system was calibrated before every examination. The results were collected after 1 and 3 h, after 24, 48, 72, and 96 h, and then, at weekly intervals for 12 consecutive weeks.

Statistical analyses were performed by Statistica v.13.3 software (Tibco Software Inc., Palo Alto, CA, USA). Descriptive data were presented as mean and standard deviation (±SD). The distribution of data was performed using the Shapiro–Wilk normality test. Differences between the daily release of fluoride in FAp were analyzed using ANOVA for dependent samples, then by a post hoc Tukey test. The Student t-test was performed for calculation of differences between two independent groups of material. A *p*-value of <0.05 was considered statistically significant.

X-Ray Diffraction (XRD) measurements were made on the X’Pert PRO X-ray diffractometer (Cu Kα1, 1.54060 Å) (PANalytical, Malvern Panalytical Ltd., Malvern, UK); FT-IR spectra (Fourier Transform Infrared) measurements were performed on a Thermo Scientific Nicolet iS50 FT-IR spectrometer equipped with an ATR module (iS50 ATR) (Waltham, MA, USA). The source of infrared radiation was a HeNe laser. Scanning electron microscope (SEM) micrographs were made on an FEI Nova NanoSEM 230 microscope (Hillsboro, OR, USA).

## 3. Results

**Figure 1:**
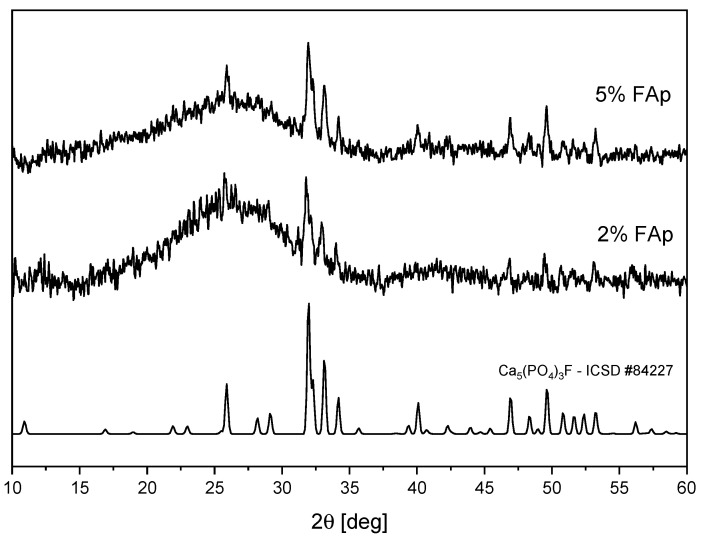
XRD diagrams of polymers with different contents of FAp.

Figure 1 shows the XRD patterns of polymers with 2% and 5% of FAp prepared in the form of pellets. For both materials, there are peaks visible, which perfectly match the theoretical pattern of Ca_5_(PO_4_)_3_F fluoroapatite (ICSD No. 84227). A broad band is also visible, which is typical for amorphous polymer matrix. For the sample with 2% of FAp, this band is stronger.

**Figure 2:**
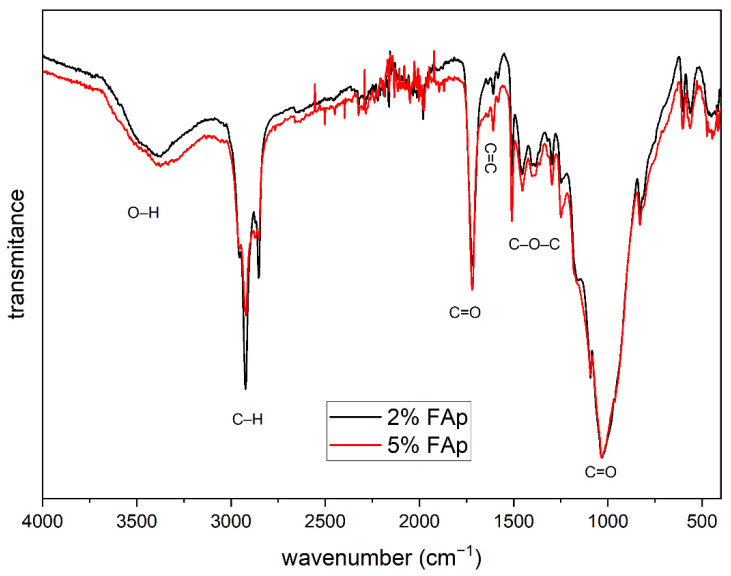
FTIR spectra of polymers with different content of FAp.

Figure 2 presents the FTIR spectrum that shows the characteristic bands for the organic polymer materials. The bands with a wavenumber from 750 to 1200 and 1725 cm^−1^ come from the vibrations of the C=O double bond. The bands from 1250 to 1500 cm^−1^ are related to the vibration of the C–O–C molecule, and the band located at 1610 cm^−1^ corresponds to the C=C double bond characteristic for dental materials, while intense bands at 2850 and 2915 cm^−1^ are related to the vibrations of the C–H bond. In addition, a weak band is visible around 3500 cm^−1^ associated with the vibration of the O–H group.

**Figure 3:**
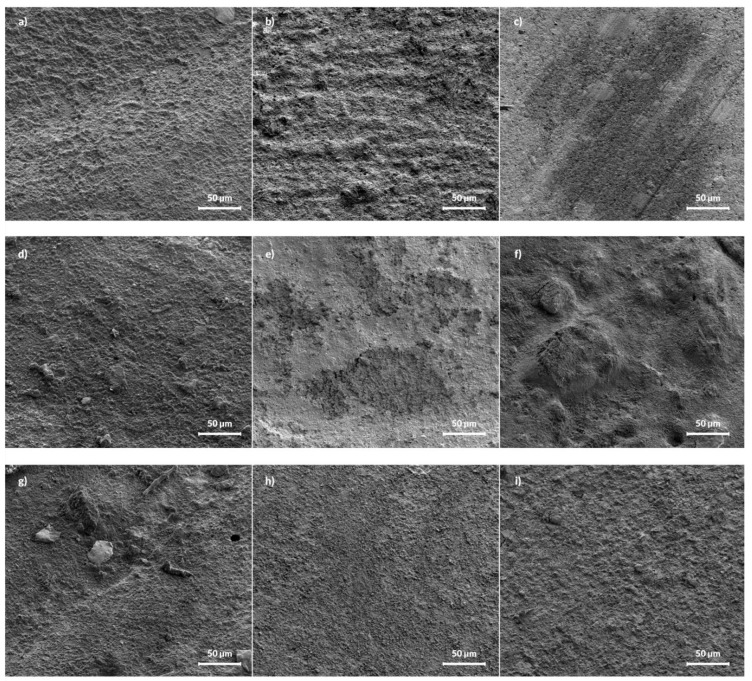
SEM images of pure i-FLOW (**a**–**c**), i-FLOW with 2%FAp (**d**–**f**), and 5%FAp (**g**–**i**) before release (left column) and after 12 weeks of release in deionized water (middle column) and 0.9% NaCl solution (right column), respectively.

The SEM micrographs of pure i-FLOW and polymer with 2 and 5%FAp are presented in Figure 3. The pure i-FLOW sample has a smooth surface, while samples with 2 and 5% FAp have some crystals visible. After release, the most significant change was visible for the sample with 2%FAp, on which the surface became more porous with visible craters. The surface of the 5%FAp sample also became porous, but there are no visible craters.

**Figure 4:**
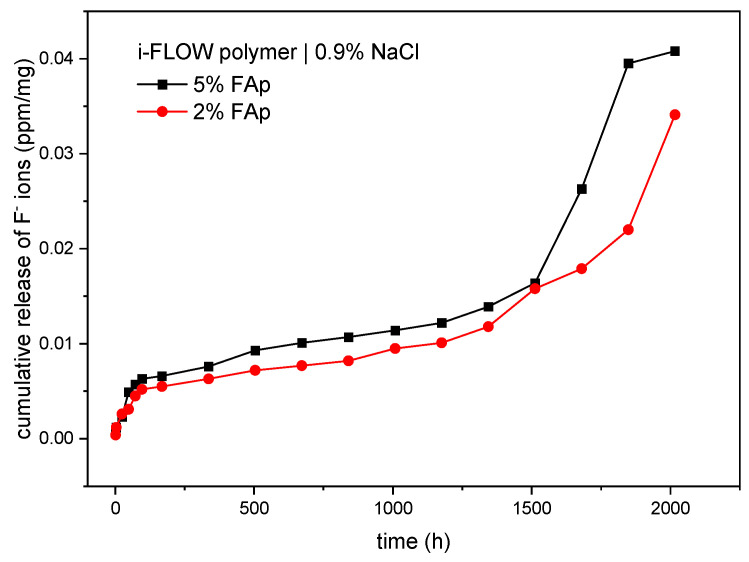
Cumulated release of fluoride ions (ppm/mg) from two study materials into 0.9% NaCl.

As displayed in Table 1, there were statistically significant differences in the time period of release of fluoride ions from the 5%FAp and 2%FAp materials into the saline solution (*p* < 0.0001). The highest fluoride release from the 5%FAp + polymer was observed in weeks 10 and 11 of incubation, and from the 2%FAp + polymer in weeks 9, 11, and 12 of incubation. The total level of fluoride ions released from the 5%FA+polymer (0.0407 ± 0.0165 ppm/mg) was similar to the 2%FAp + polymer (0.0341 ± 0.0081 ppm/mg). The mean levels of fluoride released were similar in both materials (*p* = 0.637). The cumulative release of both materials in time is presented in Figure 4.

**Figure 5:**
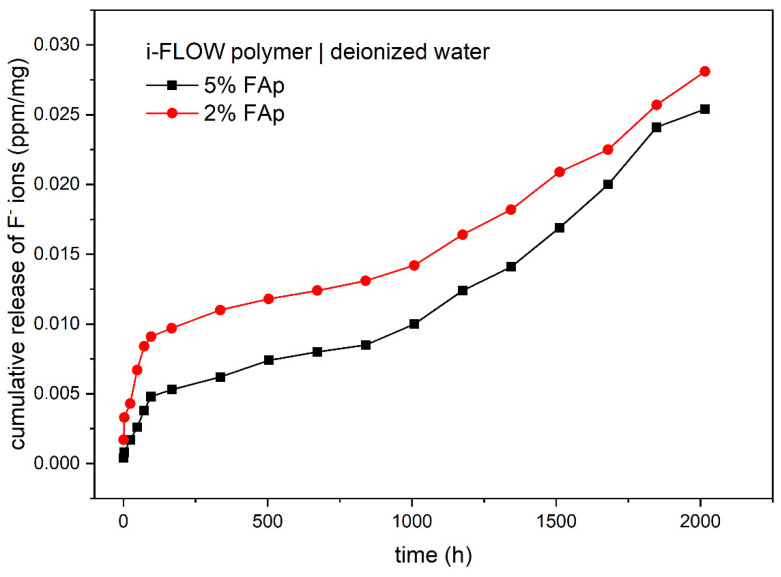
Cumulated release of fluoride ions (ppm/mg) from two study materials into deionized water.

Table 2 shows the release of fluoride ions from two study materials into deionized water. The highest concentration of fluoride ions was observed for the 5%FAp + polymer in weeks 9, 10, and 11 of incubation and for 2%FAp + polymer, in weeks 9, 11, and 12 of incubation. The time periods of the release of fluoride ions were significantly different for each of the study materials (*p* < 0.0001 or *p* = 0.0001). The 5%FAp + polymer and 2%FAp + polymer materials showed similar levels of cumulative release of fluoride ions into deionized water (0.0254 ± 0.0044 ppm/mg and 0.0281 ± 0.0060 ppm/mg, respectively). In addition, the mean levels of fluoride released from the 5%FAp + polymer and the 2%FAp + polymer were similar (*p* = 0.420). The cumulative release of both materials in time is presented in Figure 5.

Comparative analysis demonstrated a lack of significant differences in the release of fluoride ions from the 5%FAp + polymer into 0.9% NaCl or deionized water (*p* = 0.154) and from 2%FAp + polymer into the same solutions (*p* = 0.457).

## 4. Discussion

Like all fields of medicine, dentistry is constantly evolving. For centuries, focusing on the development of both new methods of treatment and new materials, scientists have been searching for materials with ever-better parameters and properties that would be able to satisfy the needs of patients and would be desirable for use in the oral cavity.

Nanomaterials are synthetic or natural materials whose components are smaller than 100 nm in at least one dimension. A great advantage of nanomaterials is that, because of their small size, they have a significantly greater surface area per mass unit compared to larger particles, which alters properties including electrical, optical, and magnetic [16,20,21,22,23,24,25]. Currently, nanotechnology is used to treat dental diseases and dentin hypersensitivity, remineralize dental tissue, and prevent biofilm formation and the progression of dental caries [24,25].

The use of nano-hydroxyapatite (HA) in the treatment of carious lesions attracts great interest. In nature, Hydroxyapatites (HA; Ca_10_(PO_4_)_6_(OH)_2_) occur as geological and biological minerals, in which case they constitute an inorganic component of bones, teeth, and geological minerals. Due to its excellent biological properties and biocompatibility (the similarity of its composition and crystalline structure to the apatite of dental enamel and the human skeletal system), HA is successfully used in various fields of dentistry, including prevention, restorative dentistry, periodontics, surgery, or implantology. Fluoroapatite is another biomedical material that is used increasingly often (FA; Ca_10_(PO_4_)_6_F_2_). FA is found in the enamel and is structurally very similar to hydroxyapatite (HA) but has a higher thermal stability, greater tolerance to acid solubility, and greater mechanical strength than HA [26,27,28,29,30,31].

It has been demonstrated that the addition of FA and HA has an impact on increasing the mechanical strength and bioactivity of glass ionomer cements while maintaining their clinical properties. This leads to the conclusion that due to their nanobioceramics content, glass ionomer cements are promising restorative materials with improved mechanical properties and better dentin bond strength [32,33,34,35].

Studies conducted by Vano et al. [36] show that the use of nano-hydroxyapatite in a fluorine-free toothpaste effectively relieves the symptoms of dentinal hypersensitivity after only 2 to 4 weeks. Wang et al. [37] and Amaechi et al. [38] also demonstrated that the effectiveness of nanohydroxyapatite in the treatment of dentinal hypersensitivity is similar to other available methods. Yaberil and Haghgoo [39] demonstrated the potential of nanohydroxyapatite to remineralize erosive lesions, observing a significant increase in the microhardness of demineralized enamel of permanent teeth after exposure to a 10% nanohydroxyapatite solution.

Marczuk-Kolada et al. [40] evaluated the release of fluorine ions (at 1, 4, 7, 14, 30, and day) of Fuji-IX clay cement and Dyract AP compomer (composite-modified polyacid) and their antimicrobial activity against *Streptococcus mutans, Streptococcus salivarius, Streptococcus sanguis,* and *Lactobacillus casei subsp. Casei.* They demonstrated that the highest emission occurred on day 7, with the release of fluorine being greater in the case of Fuji-IX. Twenty-four hours after polymerization, bacterial growth in samples from Fuji-IX was inhibited, while no similar activity was observed in the case of Dyract AP; on the eighth day following polymerization, Dyract AP was significantly more active against *Streptococcus sanguis* and *salivarius*. The authors of the study emphasize the effectiveness of fluorine-releasing materials against secondary caries.

After conducting an experiment in which they observed an inhibition of in vitro demineralization around the test materials, Dionysopoulos et al. [41] also demonstrated that fluoride-releasing light-curing materials were helpful in the treatment of secondary caries.

The amount of the released fluorine ions depends on many factors, including material composition, filler type, solution type, replacement frequency, saliva composition and pH, matrix/filler ratio, material mixing procedure, irradiation time, and solution contact time, as well as solution pH and incubation time [42,43]. Garoushi et al. [44] conducted a comparative analysis of fluorine ion release by five materials with respect to their physicochemical properties and structure. They observed that different release of fluorine by each of the tested materials stemmed from their different microstructure, which was confirmed by the SEM analysis. Nigam et al. [45] observed that pH had a significant impact on the release of fluorine from filling materials, as in their study most fluoride ions were released into a pH-cycling solution. Comparing fluorine release from three different materials—glass ionomer cement (3M Ketac Molar, Saint Paul, MN, USA), resin-modified glass ionomer cement (ACTIVA BioActive-Restorative, Pulpdent, Watertwon, MA, USA), and nanocomposite (Tetric EvoCeram, Ivoclar Vivadent AG, Schaan, Liechtenstein)—to various environments (deionized water, artificial saliva, and pH-cycling solution), Porenczuk et al. [46] observed the greatest fluorine emission in pH-cycling solution and the lowest in artificial saliva. The greatest release of fluorine was observed in the case of the glass ionomer and the lowest in the case of the nanocomposite, with a significant difference found between these materials.

The authors’ own studies revealed statistically significant differences in the periods of the release of fluoride ions from 5%FAp and 2%FAp materials into saline solution as well as into deionized water. The highest fluorine release from a 5%FAp + polymer was observed in the 10th and 11th week of incubation (for saline solution), in the 9th, 10th, and 11th week (for deionized water), and from a 2%FAp + polymer in the 9th, 11th, and 12th week of incubation (for both environments). Similar results were obtained by Mystkowska [47] in her study. She compared fluorine release from three different composites: composite A, containing 60% vol. of fluorinated J-20 glass; composite B, with an addition of J-20 glass and strontium fluoride; and composite C, which included nanosilica in addition to both fluorine sources. She demonstrated that the composite based on fluorinated J-20 glass released the most fluorine, and the amount of the released fluorine increased until day 7 of the study, after which it stabilized. The author concludes that fluorinated glass is the main source of fluoride ions. The lowest emission of these ions was observed in the case of the composite blended with strontium fluoride and nanosilica. Interestingly, however, it was accompanied by a continuous increase in the release of fluorine ions into the contact solution. According to the author, it can be explained by the lower total content of fluorine in the structure of the composite as well the fact that the free spaces between the particles of fluorinated glass and strontium fluoride are occupied by small grains of nanosilica, which block the path of the fluorine ion’s diffusion into the environment.

According to Kosior et al. [48], the greatest release of fluoride ions for the various materials tested occurred on days 49, 56, 70, and 77 of the study. On the other hand, Nigam et al. [45] and Gui et al. [49] observed that the release of fluorine ions was the greatest on the first day of the experiment, with the highest emission from glass ionomer cements and the lowest from composites. In our study, we observed that the total level of fluoride ion release from the 5%FAp + polymer was similar to the 2%FAp + polymer in both tested environments. The mean levels of fluoride release were also similar in both materials.

Two different mechanisms are responsible for the release of fluoride ions from prepared samples. The first is caused by dissolution of surface material and the second is diffusion caused by the appropriate counter ion such as sodium or by exchange of hydroxyl groups in an aqueous environment [45,50,51]. According to cumulative release shown in Figure 4 and Figure 5, the dissolution is visible at the beginning of the process in both environments and after 1500 h in 0.9% NaCl solution. It is manifested by a fast increase in fluoride ions in the solution. The diffusion is responsible for the rest of the process. It is also confirmed in the SEM images (Figure 3), in which the disappearance of FAp nanocrystals from the surface is visible.

## 5. Conclusions

Both tested materials (5%FAp and 2%FAp) exhibited the ability to release fluoride ions over long periods of time under experimental conditions, and the levels of the released ions differed significantly in specific time intervals. The processes responsible for fluoride release are dissolution and the diffusion of ions.

Further studies concerning the practical use of these new, original nanomaterials in restorative dentistry should be conducted.

## Figures and Tables

**Table 1:** Release of fluoride ions (ppm/mg) from 5%FAp + polymer and 2%FAp + polymer materials into saline solution (0.9% NaCl).

Time	5%FAp + Polymer (ppm/mg) Mean ± SD	2%FAp + Polymer (ppm/mg) Mean ± SD
1 h	0.0007 ± 0.0006	0.0004 ± 0.0001
3 h	0.0005 ± 0.0002	0.0008 ± 0.0002
24 h	0.0011 ± 0.0003	0.0014 ± 0.0003
48 h	0.0026 ± 0.0010	0.0005 ± 0.0001
72 h	0.0008 ± 0.0004	0.0014 ± 0.0001
96 h	0.0006 ± 0.0002	0.0007 ± 0.0001
1 week	0.0003 ± 0.0001	0.0003 ± 0.0001
2 weeks	0.0010 ± 0.0002	0.0008 ± 0.0001
3 weeks	0.0017 ± 0.0008	0.0009 ± 0.0002
4 weeks	0.0008 ± 0.0003	0.0005 ± 0.0001
5 weeks	0.0006 ± 0.0002	0.0005 ± 0.0001
6 weeks	0.0007 ± 0.0002	0.0013 ± 0.0004
7 weeks	0.0008 ± 0.0005	0.0006 ± 0.0001
8 weeks	0.0017 ± 0.0001	0.0017 ± 0.0001
9 weeks	0.0025 ± 0.0006	0.0040 ± 0.0011
10 weeks	0.0099 ± 0.0009	0.0021 ± 0.0005
11 weeks	0.0132 ± 0.0109	0.0041 ± 0.0008
12 weeks	0.0013 ± 0.0001	0.0121 ± 0.0063
Cumulative release of F—ions (ppm/mg)	0.0407 ± 0.0165	0.0341 ± 0.0081
*p*-value (ANOVA for dependent samples)	<0.0001	<0.0001
Mean ± SD	0.0023 ± 0.0014	0.0019 ± 0.0013
5%FAp + polymer vs. 2%FAp + polymer (Student-*t* test)	0.637	

**Table 2:** Release of fluoride ions (ppm/mg) from 5%FAp + polymer and 2%FAp + polymer materials into deionized water.

Time	5%FAp + Polymer (ppm/mg) Mean ± SD	2%FAp + Polymer (ppm/mg) Mean ± SD
1 h	0.0004 ± 0.0001	0.0017 ± 0.0002
3 h	0.0004 ± 0.0001	0.0016 ± 0.0006
24 h	0.0009 ± 0.0005	0.0010 ± 0.0007
48 h	0.0009 ± 0.0004	0.0024 ± 0.0003
72 h	0.0012 ± 0.0001	0.0017 ± 0.0004
96 h	0.0010 ± 0.0005	0.0007 ± 0.0003
1 week	0.0005 ± 0.0001	0.0006 ± 0.0002
2 weeks	0.0009 ± 0.0002	0.0013 ± 0.0010
3 weeks	0.0012 ± 0.0001	0.0008 ± 0.0001
4 weeks	0.0006 ± 0.0001	0.0006 ± 0.0001
5 weeks	0.0005 ± 0.0001	0.0007 ± 0.0004
6 weeks	0.0015 ± 0.0004	0.0011 ± 0.0001
7 weeks	0.0024 ± 0.0006	0.0022 ± 0.0001
8 weeks	0.0017 ± 0.0002	0.0018 ± 0.0004
9 weeks	0.0028 ± 0.0006	0.0027 ± 0.0005
10 weeks	0.0031 ± 0.0014	0.0016 ± 0.0001
11 weeks	0.0041 ± 0.0003	0.0032 ± 0.0003
12 weeks	0.0013 ± 0.0001	0.0024 ± 0.0008
Cumulative release of F^-^ ions (ppm/mg)	0.0254 ± 0.0044	0.0281 ± 0.0060
*p*-value (ANOVA for dependent samples)	<0.0001	0.0001
Mean ± SD	0.0014 ± 0.0011	0.0016 ± 0.0009
5%FAp + polymer vs. 2%FAp + polymer (Student-*t* test)	0.420	

## Data Availability

Not applicable.
